# Understanding the Purchasing and Consumption Dynamics of Commercially Processed Complementary Foods and Caregiver Motivations and Reasons for Purchasing These Foods in Nairobi

**DOI:** 10.1111/mcn.70102

**Published:** 2025-09-07

**Authors:** Antonina N. Mutoro, Maureen Gitagia, Charity Zvandaziva, Veronica Sanda Ojiambo, Gershim Asiki, Elizabeth Kimani‐Murage

**Affiliations:** ^1^ African Population and Health Research Center Nairobi Kenya; ^2^ UNICEF Kenya Nairobi Kenya

## Abstract

Commercially processed complementary foods (CPCFs) are consumed in Kenya, but little is known about caregiver perceptions and reasons for their consumption. We explored caregiver perceptions, motivations and reasons for purchasing CPCFs. This cross‐sectional mixed‐methods study was conducted in Nairobi among caregivers of children aged 6–23 months. A four‐stage sampling strategy was used to select study sites non‐slum (Westlands) and slum (Mathare) areas, retail outlets and study participants. Eighty‐one caregivers (40 in Mathare, 41 in Westlands) were recruited for the quantitative survey, from this sample 16 participants were recruited for qualitative in‐depth interviews. Questions about the place of purchase, types of foods purchased, reasons for purchase, sources of information on infant and young child feeding and CPCFs, and perceptions on health and nutrition claims were asked. Nearly all caregivers (96.3%) reported giving their children CPCFs. Close to half of caregivers offered CPCFs as a snack (46.9%) while 21% offered them as a main meal. CPCFs were perceived to be healthy (73.1%), nutritious (71.8%) and easy to prepare (70.7%) and child preference (55.6%), price (54.3%), taste (51.9%), nutritional quality (55.6%) and food safety (62.9%) were considered important factors when purchasing them. Nutrition and health claims on product packaging were documented, and these appeared to influence caregiver perceptions about CPCFs. Participants perceived CPCFs as good and healthy for children and rich in nutrients essential for growth and development. This is in line with nutrition claims on these products. CPCFs are highly regarded by caregivers and are consumed in slum and non‐slum settings in Nairobi. Given that misleading health and nutrition claims are used to market them, CPCFs may negatively impact child health if their marketing and consumption are not regulated.

## Summary

- Commercially processed complementary foods are relatively common in urban slum and non‐slum areas and are perceived to be healthy, safe and nutritious.
- Caregivers use food labels to make purchasing decisions around CPCFs and the health and nutrition claims presented on the labels influence their perceptions about the benefits of the CPCFs.
- There is a need for regulation on their composition and the use of health and nutrition claims on these products as the claims can be misleading.

## 1 Background

The barriers to meeting infants and young children's nutrient needs are uniquely challenging in Kenya and Africa at large. As more families move to cities, their diets are constrained due to poverty, inequities, and the increasing cost of nutritious food. More women are participating in the workforce, often while continuing to carry the greatest burden of caregiving and household duties, restricting the time they have to prepare healthy homemade foods. Consequently, there is a shift from traditional diets towards convenient processed foods, including commercially processed complementary foods (CPCFs) which are marketed to older infants and young children aged 6–23 months (Stuckler and Nestle 2012; Baker et al. 2021).

CPCFs are described as pre‐packaged food and beverage products which require minimal to no cooking or heating before consumption (Maslin and Venter 2017). These include a broad range of products, wet (e.g., purees) or dry (e.g., cereals), that are marketed for complementary feeding to older infants and children between 6 and 36 months (Debessa et al. 2023). CPCFs were classified into different categories including dry and instant cereals, dry finger foods, ready‐to‐eat purees/meals including fruit and vegetable purees and juices and other drinks, dairy products. CPCFs are often higher in salt, sugar and unhealthy fat, and low in essential micronutrients (World Health Organization 2019). Little is known about the consumption of CPCF in Africa, yet the CPCF market is dynamic with an increasing number of small, local producers entering the existing multinational‐dominated market (Reardon et al. 2021; Abizari et al. 2017; Debessa et al. 2023). Despite the increased demand for CPCFs in Africa, there is a lack of regulations on their nutrient composition that is the maximum acceptable amount of sugar, salt and unhealthy fat and the minimum acceptable amounts of vitamins and minerals (Aryeetey and Tay 2015; Khosravi et al. 2023). Studies in European countries show that CPCFs tend to be high in sugar, fat and salt (Hutchinson et al. 2020), but there is limited evidence on their nutrient composition in Africa.

The WHO Guidance on Ending the Inappropriate Promotion of Foods for Infants and Young Children, included as part of WHA 69.9, recommends that Nutrient profile models (NPMs) should be developed and utilized to guide decisions on which foods are inappropriate for promotion (World Health Organization 2019). This recommendation assumed that national standards for CPCFs would be developed to define the requirements of unhealthy or healthy CPCFs. While this recommendation is critical to the guidance, currently no country in Africa has compositional standards for CPCFs, and this component of the recommendation has been largely redundant.

A Kenyan NPM has been developed, but it does not account for nutrient thresholds for children below 36 months, and it excludes foods specifically manufactured for infants and young children (Ministry of Health 2025). The KNPM has 11 food categories some of which could potentially cover foods consumed by children aged between 6 and 36 months including fruits and vegetables which considers purees and pulps; dairy products and analogues; composite foods, beverages and cereal and cereal products. Taking nutrient thresholds into account for children aged 6–36 months and inclusion of products targeting this age group will ensure that parents and other caregivers receive clear and accurate information on the best way to feed their infants and young children, as well as aid in the regulation of health and nutrition claims.

Unhealthy products frequently use unregulated health and nutrition content claims and unregulated wellness messaging, leading to consumers believing these products are healthier and more nutritious for children. According to an online survey of U.S. parents, the majority of them choose their children's food and drinks based on the ingredients or claims on the product packaging; parents prefer drinks with low‐calorie and natural claims (C.R. Munsell et al. 2015). According to one study, parents perceived products with health or nutrition claims to be more nutritious for their children (Koo et al. 2018). Understanding how caregivers perceive nutrition and health claims when purchasing foods for their infants and young children is critical for informing future regulatory guidelines for nutrient profile models for complementary feeding.

This study aimed to understand caregiver perceptions and practices in relation to purchasing CPCFs for their children. We assessed the purchasing and consumption dynamics of CPCFs including the frequency, place of purchase, types of products purchased and use of products; mapped and described the sources of information for caregivers on infant and young child feeding; explored caregiver motivations and reasons for purchasing CPCFs and caregivers' perception of nutrition and health claims and other labels and how this influences their purchasing decisions. We also documented the types of marketing strategies used in stores and on product packaging of CPCFs.

## 2 Methodology

### 2.1 Study Setting and Design

The study was conducted in two settings in Nairobi in 2023: Westlands, a high socioeconomic area and Mathare, an informal settlement in a low socioeconomic area. The two sites were selected based on government multidimensional poverty indices provided by the Kenya National Bureau of Statistics (Kenya National Bureau of Statistics, KNBS 2023). Nairobi, the capital city of Kenya is home to approximately 4.9 million people over half of whom reside in informal settlements. According to the 2022 Kenya Demographic and Health Survey profile for Nairobi City County, 11% of children under 5 years are stunted which is lower than the national prevalence (18%) but this varies based on location as stunting rates close to 50% have been reported in informal settlements in the city (KNBS and ICF 2023; Kimani‐Murage et al. 2015). Additionally, the prevalence of wasting and underweight in Nairobi is 3% and 5%, respectively ‐ both lower than the national averages of 5% and 10%. Of concern, however, is the overweight prevalence of 6%, which is double the national average of 3%. This was a cross‐sectional study which used a triangulation design which involved the simultaneous collection of both qualitative and quantitative data to give a description of caregiver use and perceptions around CPCFs. Table S1 shows a summary of data collection methods by objective.

### 2.2 Sampling Strategy

**Figure 1:**
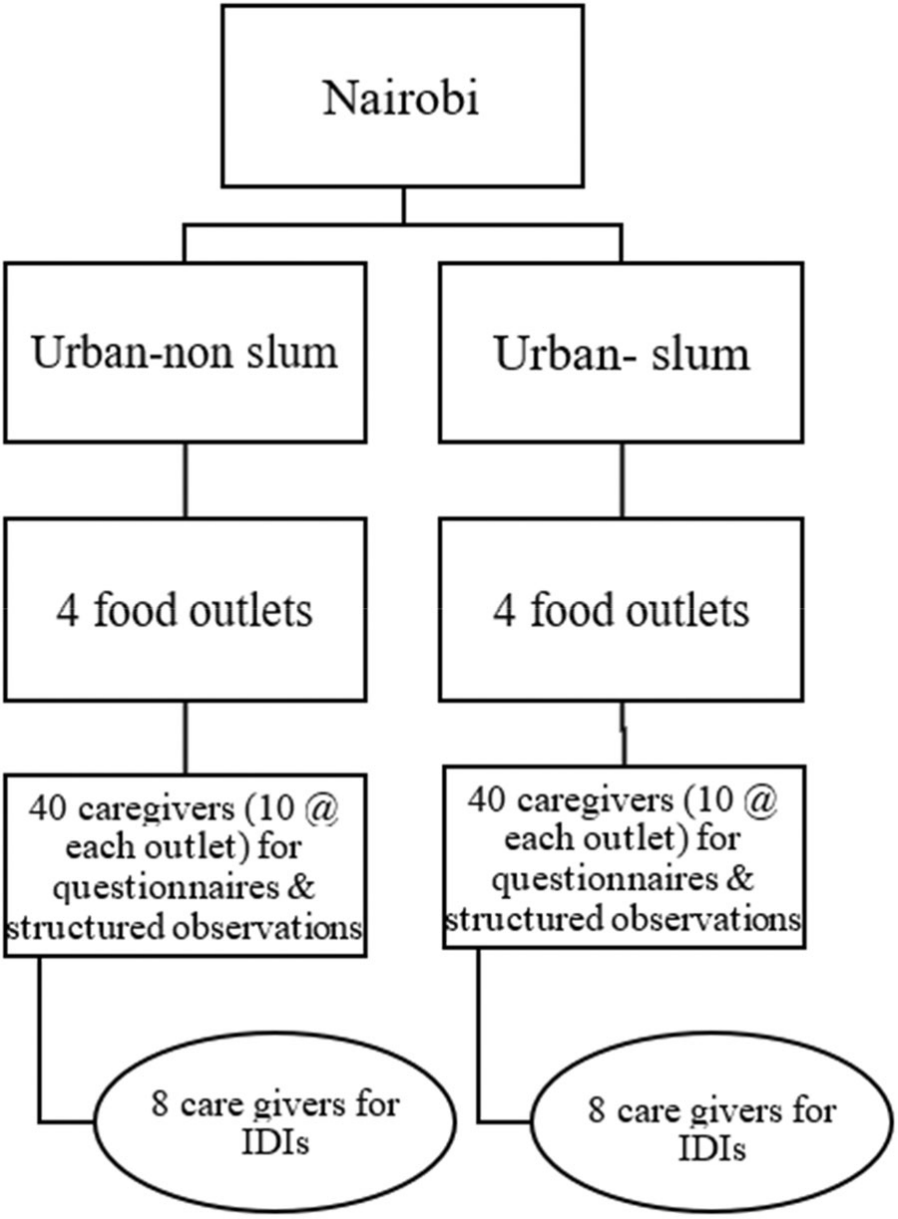
Four‐stage sampling scheme for the study.

The sampling scheme was a four‐stage sampling using a combination of purposive and random sampling (Figure 1). In Stage 1, Nairobi County was purposively selected to represent urban areas because of logistical reasons, and CPCFs are commonly sold and consumed in this setting. In Stage 2, Nairobi was stratified into slum (Mathare) and non‐slum (Westlands) sub‐counties, and one sub‐county from each stratum was selected. We had earlier completed the mapping of all retail food outlets in Nairobi, stratified into urban‐non‐slum and slum. These provided a sampling frame for the 3rd stage, which involved random selection of four medium or large retail outlets from each sub‐county (urban non‐slum and slum). In stage four, 10 caregivers to children aged between 6 and 23 months were consecutively recruited as they exited the selected retail food outlets at the cashpoint (in total 80). Only primary caregivers (mothers and fathers), who met the inclusion criteria and were willing to participate in the study were interviewed. To ensure diversity and relevance of participant perspectives, we used a purposive sampling approach. A sub‐sample of caregivers who had already participated in the quantitative cross‐sectional survey was invited to take part in the qualitative interviews. Specifically, we recruited two participants per retail outlet, selecting the 5th and 10th survey participants at each location. This systematic selection strategy was designed to ensure a consistent and transparent recruitment process while capturing variation across respondents. Participants were approached directly after completing the survey and invited to participate in an in‐depth interview (IDI), with verbal and written informed consent obtained before participation.

### 2.3 Data Collection

A list of research tools, including the International Network for Food and Obesity/Non‐communicable Diseases Research, Monitoring and Action Support (INFORMAS) tools have been developed (Swinburn et al. 2013). These tools have been adapted to capture double‐duty actions in the retail food environment assessments in Kenya, Tanzania and Uganda. We included select questions from the tools to capture indicators for CPCF assessments in food outlets, including types of in‐store advertisements, location of CPCFs in the store and their placement on shelves. We also documented information on product packaging, including marketing strategies used, nutrient composition and the use of health and nutrition claims.

The data collection activities were undertaken by contracted research assistants who underwent a 5‐day training workshop led by the study team. During this workshop, the research assistants were trained in the ethics of conducting research involving human participants and in the use of study tools. During this period, all the study tools were piloted and refined based on participant feedback to ensure clarity and accuracy of the data captured.

The survey estimated how often food products were purchased and identified the place of purchase, types of products and the use of products by caregivers in urban contexts in Kenya. It also captured the sources of information for caregivers on young infant feeding.

In‐store observations were made in each retail outlet using a checklist including information on types of CPCFs, marketing strategies used in store and on product packaging and nutrition composition information on food packaging. Products were selected if they specifically targeted children aged between 6 and 23 months as indicated by the age of consumers on the package and words such as weaning or complementary feeding.

We collected qualitative data through in‐depth interviews (IDIs) guided by semi‐structured interview tools developed by the research team. The guide was aligned with the study's objectives and grounded in a phenomenological approach, enabling us to explore how participants experienced and interpreted issues in their own words. The interview guide focused on caregivers' motivations for purchasing commercially produced complementary foods (CPCFs), their perceptions of nutrition and health claims, and how labelling influenced their purchasing decisions.

Interviews lasted between 45 min to 1 h. To ensure consistency and rigour in data collection, interviewers completed a five‐day training covering ethical practices, qualitative interviewing techniques, study objectives, and use of the guide. The tool was pilot tested and refined based on feedback from both the field team and the broader research group to enhance clarity and contextual relevance.

All interviews were conducted in the local language and subsequently translated and transcribed verbatim into English by a trained bilingual transcriber. Transcripts were reviewed for accuracy and fidelity by the research team to ensure they accurately reflected participants' original meanings and expressions.

### 2.4 Data Processing and Analysis

Data collected using Likert scales was recoded to summarize responses. The level of importance was recoded from totally important, important, neutral, unimportant, totally unimportant to important, neutral and unimportant. Similarly, the level of agreeableness was recorded from totally agree, agree, neutral, disagree, totally disagree to agree, neutral and disagree. Descriptive analysis was conducted to estimate the frequency of demographic characteristics, place of purchases, types of products purchased, and the use of products expressed as percentages. Sources of information from respondents on CPCFs and marketing strategies used to promote CPCFs were also summarized using frequencies and percentages. Pearson chi‐squared correlation test at 5% was used to compare categorical variables by location (urban non‐slum and urban slum). To estimate the key factors that caregivers considered when purchasing CPCFs, descriptive analysis was used to estimate the proportion of caregivers who either strongly agreed or considered some factors as very important when purchasing CPCFs. Variables with the highest proportions of respondents were considered as key. Data obtained from in‐store observations was summarized in text form, given that few products were listed.

The qualitative data were translated and transcribed verbatim into English for analysis. We used Braun and Clarke's (2006) thematic analysis framework to guide our qualitative data analysis. Two researchers independently led the coding process. One undertook manual coding using printed transcripts and thematic note‐taking, while the other used NVivo 12 software to code the same data set digitally. The researchers held consensus meetings to compare, discuss and reconcile differences in their coding. This dual approach allowed for comparison between coding methods and cross‐validation of interpretations, strengthening the consistency and reliability of the findings.

An initial codebook was developed from a subset of transcripts and refined iteratively through team discussions. Discrepancies in coding were resolved through reflection and dialogue, ensuring alignment across the team while remaining open to emerging insights from the data. Themes were identified by grouping related codes and identifying patterns that addressed the core research questions. Analysis continued until thematic saturation was achieved.

Emerging themes were then synthesized in relation to caregiver motivations and reasons for purchasing commercially produced complementary foods (CPCFs), along with their perceptions of nutrition and health claims and other labelling information, and how these factors influenced their purchasing decisions. Nutrition claims were defined as any claim, which states, suggests or implies that a food has particular beneficial nutritional properties due to the energy (caloric value) it provides at a reduced or increased rate or does not provide (European Union Regulation 2006). The definition also included the nutrients or other substances it contains in reduced or increased proportions or does not contain. Health claims were defined as any statement about the relationship between food and health (European Union Regulation 2006).

### 2.5 Ethics Statement

Ethics approval for this study was obtained from the AMREF ethics scientific review committee reference number: P1256‐2022.

## 3 Results

A total of 81 participants were recruited, 41 in Westlands, 40 in Mathare, from small and medium supermarkets. Nearly all the participants were female (95%), the child's mother, with an average age of 26 years, and the average age of their children was 13 months. A summary of caregiver characteristics is presented in Table 1. Participants in Mathare were more likely to be casual labourers, while participants recruited in Westlands were more likely to be employed. Participants in Westlands were also more likely to be married than participants in Mathare.

**Table 1:** Characteristics of caregivers of children aged 6–23 months from Mathare and Westlands (*n* = 81).

Characteristic	Total (%)	Mathare (%)	Westlands (%)	*p*‐value
**Education level**				
**Female**	95.0	46.9	48.1	—
Primary school	17.3	12.5	22.0	0.36
Secondary school	56.8	57.5	56.1	
College/University	23.5	25.0	21.9	
Vocational training	2.5	5.0	0	
**Religion**				
Christian	93.8	90.0	97.6	0.07
Muslim	4.9	10.0	0	
Not willing to answer	1.2	0	2.4	
**Marital status**				
Currently married	54.3	37.5	70.3	0.009
Living together	6.2	12.5	0	
Separated	8.6	15.0	2.4	
Divorced	2.5	2.5	2.4	
Never married	28.4	32.5	24.4	
**Spouse's education level (*n*** = **49)**				
Primary school	12.4	5.0	17.2	0.21
Secondary school	59.2	70.0	51.7	
College/University	26.5	20.0	31.0	
Vocational training	2.6	5.0	0	
**Source of income**				
Employee	27.2	2.5	51.2	< 0.001
Entrepreneur	14.8	20.0	9.8	
Unemployed	34.6	32.5	36.6	
Casual labourer	23.5	45.0	2.4	

Nearly all participants in both study sites reported purchasing dry, powdered, and instant cereal/starchy and breakfast cereals (96.3%). Juices and other drinks were purchased by one‐third of the participants. Close to half the (46.9%) participants reported giving CPCFs as a snack, while 21% reported giving it as a main meal.

**Figure 2:**
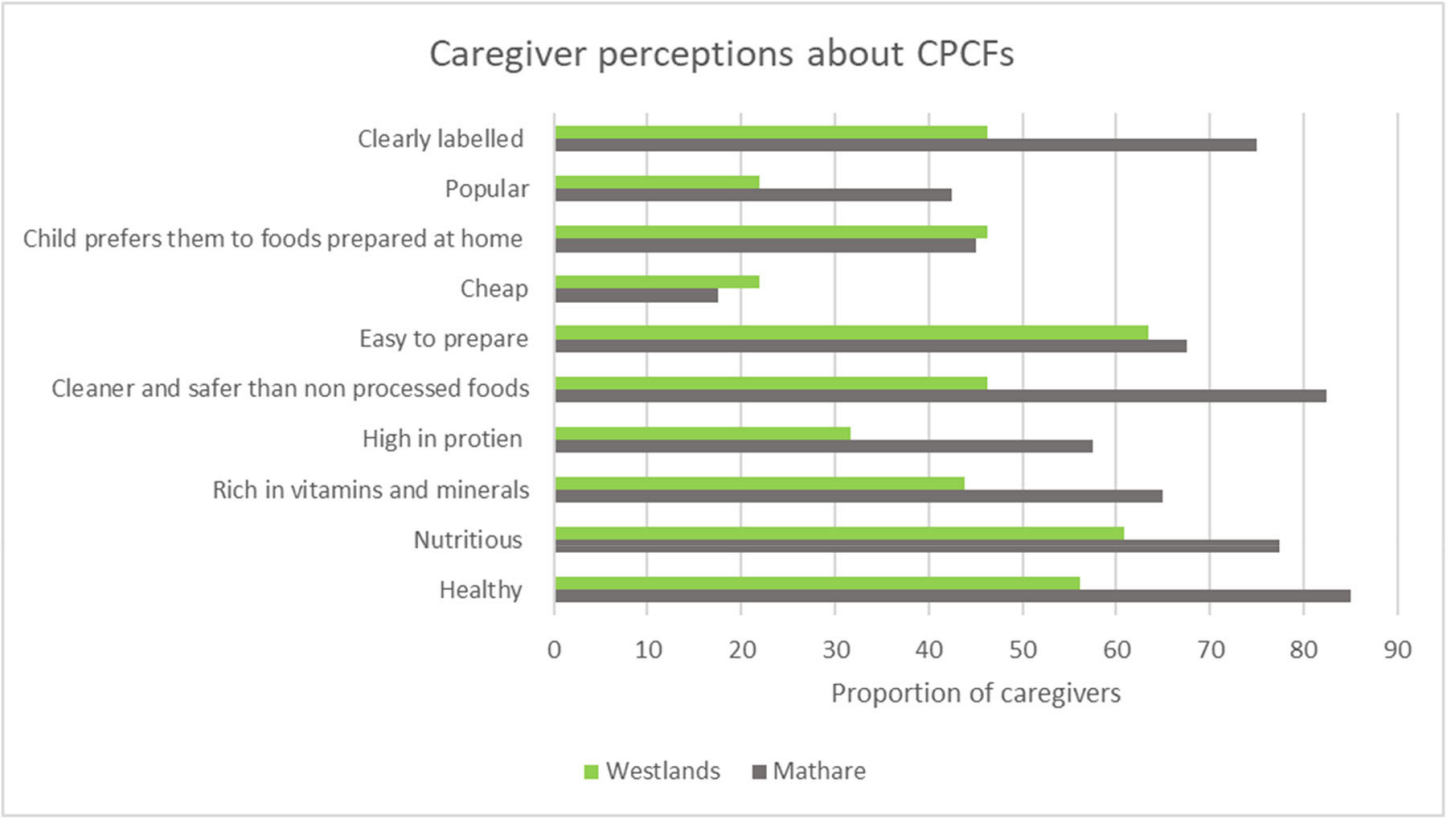
Caregiver perceptions about CPCFs – Factors they strongly agree with by location.

Table S2 shows a summary of the characteristics of CPCFs rated by caregivers. A large proportion of participants agreed that CPCFs are healthy, nutritious, contain vitamins and minerals, are high in protein, are cleaner and safer than non‐processed foods, are easy to prepare, and are clearly labelled (Table S2). A smaller proportion of participants agreed that they were cheap, preferred by their children, and popular. There was no difference in responses by study location. We assessed the characteristics of CPCFs that caregivers strongly agreed with to find out which was the most popular characteristic. Over 70% of participants strongly agreed that CPCFs were healthy, nutritious, and easy to prepare (Figure S1). Compared to caregivers in Westlands, caregivers in Mathare were more likely to strongly agree that CPCFs are healthy, nutritious, clearly labelled, popular, cleaner and safer than non‐processed foods (46.3% vs. 82.5% *p* = 0.012) (Figure 2). CPCFs were also perceived to prevent diseases, promote child health, growth and development because of the nutrients they contain.

…they benefit my child because they are rich in vitamins so when they consume them, they have good health.

–IDI: Mother, 21 years, Mathare

I have a feeling and thoughts that these foods are okay and you can actually get them packaged even in small quantities so that any customer can be able to afford. Also, they are hygienically stored and so far my child hasn't developed any complications as a result of consuming these CPCFs. They also have nutrients and every vital component required by children.

–IDI: Mother,23 Years, Westlands

**Figure 3:**
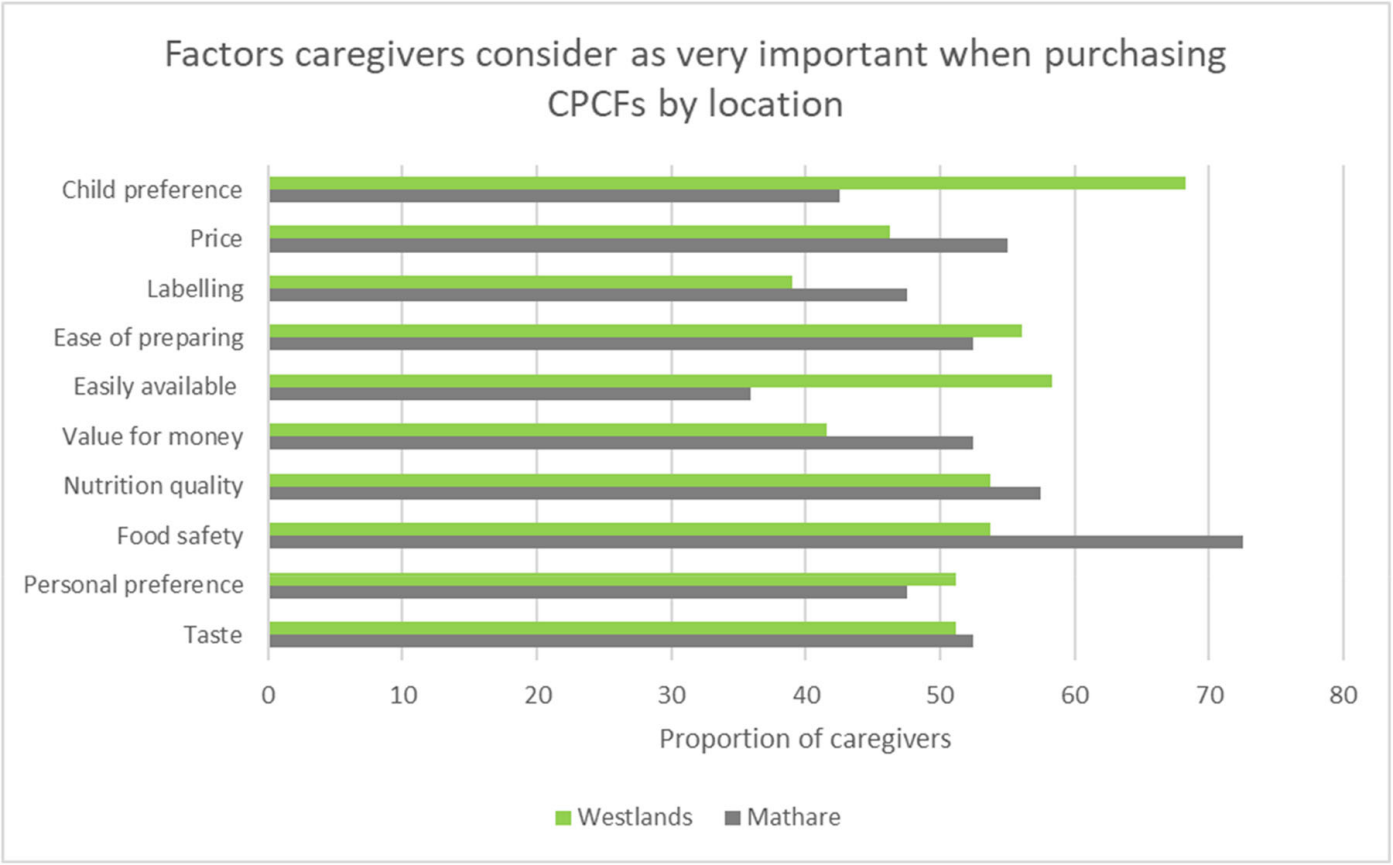
Factors caregivers considered very important when purchasing CPCFs by location.

Table S4 shows a summary of factors that caregivers consider to be important when purchasing CPCFs. All participants highlighted that food safety was important for them. Personal preference, nutrition quality, value for money, availability, ease of preparation, labelling, price was also considered important. There were no differences in responses by location for all the above factors apart from child preference. Caregivers in Westlands were more likely to rate child preference as important compared to caregivers in Mathare (95.6% vs. 70% *p* = 0.004). Reasons considered as very important by more than 50% of participants included: child preference, price, ease of preparation, nutrition quality, food safety and taste (Figure S2). Compared to caregivers in Westlands, caregivers in Mathare were more likely to rate food safety, nutrition quality, value for money, labelling and price as very important factors (Figure 3).

In terms of nutrition quality, CPCFs were perceived to contain vitamins that increase the child's immunity, carbohydrates that provide energy and allow the child to play, and proteins that aid in the growth and development of children. They were also considered safe because they are packaged to prevent contamination, they do not include chemicals and additives, they have an expiry date on their packaging, and they include recipes on how to prepare the product. However, there were recommendations to the government around the involvement of the Kenya Bureau of Standards (KEBS) in checking the quality of CPCFs and confirming whether the information on packaging is correct. This was because of the potential of producers tampering with the expiry date, which may mislead consumers into purchasing the expired products.

…I think they give my child vitamins, carbohydrates and energy…Okay, for example, the vitamins boost the child's immunity, and carbohydrates give the child energy and make them strong enough to play. The proteins help with child growth and development. Although those are added proteins, they help a lot in child development both immunity and physically…

–IDI: Mother, 28 years, Mathare

My general comment is that these foods are okay, they are clean and safe from harmful substances, and they also have an expiry date on their packaging material and also have guiding instructions on how to prepare the product. The instructions also provide you with knowledge of how to use the product.

–IDI: Mother, 23 years, Westlands

**…**In my opinion, the government should be strict with those foods because, when a food is processed and already packed, if the food is spoiled or expired before packaging but they indicate a different date on the package while buying you will think the food is good but it is not. So, I think the government should be very keen or they can even involve KEBS who can check the quality of the food being packaged or confirm the information written on the package. But so far, I haven't had any issues with the food…

–IDI: Mother, 28 years, Mathare

Apart from the price of CPCFs caregivers also considered the expiration date, the manufacturer and the product packaging, including if the package is intact or the seals are broken and ingredients.

…I look out for the ingredients and the expiry dates… Maybe the price. I also look out for the price. I buy CPCFs that are affordable on my side.

–IDI: Mother, 25 years, Westlands

I do consider the food package because this is food and the package should be well sealed and not open so that the food remains clean and not contaminated.

–IDI: Mother, 28 years, Mathare

### 3.1 Sources of Information on Complementary Feeding and Commercially Processed Complementary Foods

**Figure 4:**
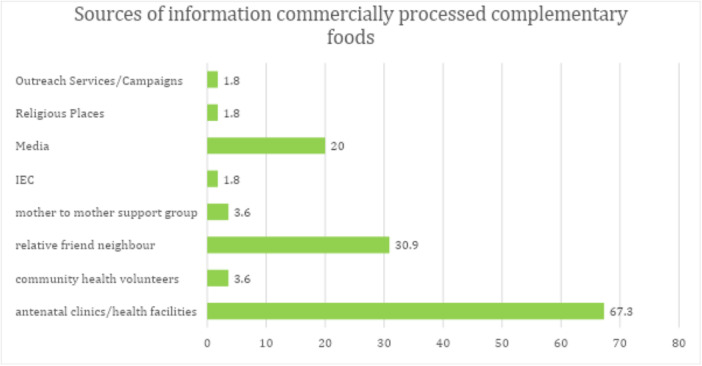
Sources of information on commercially processed complementary foods (*n* = 55).

Nearly all caregivers (91.4%) reported getting information on complementary feeding in general from antenatal clinics/health facilities (Figure S3). Antenatal clinic/health facilities were also the most trusted source of information on complementary feeding. The most common source of information on commercially processed complementary foods was antenatal clinics (67.3%) followed by relatives/friends (30.9%) and media that is television and radio (Figure 4).

Close to half of caregivers (41.8%) reported hearing/seeing advertisements for commercially processed complementary foods. The most common type of promotion reported by caregivers was price reductions (65%) and additional gifts (34.8%). Other forms of promotions/advertisements reported included entertainers/animators (8.7%), price comparison with other stores (8.7%), promotion on product packaging (8.7%) and special exhibitions on shelves.

### 3.2 In‐Store Observations

Four products targeting children aged 6–23 months were identified from the stores; three were porridge flours one was an instant cereal. The products were marketed to children aged 6 months and above and included words such as kiddos, toto and baby weaning signalling that they were targeting children. The products did not contain added sugar, salt or fat.

#### 3.2.1 Health and Nutrition Claims

Two out of the four products had nutrition claims on the front and back of the product package. The claims included Enriched with vitamins, source of energy protein and minerals, Iron, less sugar, 11+ vitamins and minerals, vitamin A + C plus zinc.

All four products included health claims on the product package, two on the back and two on the sides. Examples of health claims used included: ‘11 vitamins and minerals for healthy growth’, ‘iron for cognitive and brain development’, ‘Protein helps in the growth and maintenance of tissue, boosting immunity and providing the structure of the skin, hair, nails and tendons’.

### 3.3 Food Labels: Uses and Perceptions

Caregivers reported using food labels to check the ingredients including allergens, the nutrient content and the benefits of the nutrients for the child's growth and health and the expiration date. They also reported using the labels to obtain instructions on how to properly prepare CPCFs.

…Yes …I usually look at the content of the food, the ingredients and the nutrients in the food, the amounts and importance of the nutrients…I also check for the expiration date, allergens indicated because say my child is lactose intolerant, I will check whether a product has any milk or milk product added to it before I buy…

–IDI: Mother, 28 years, Mathare

…Yes. The information on the food labels helps me to make purchase decisions. It also informs me with the nutrition information of the product e.g. the food has iron, proteins and or carbohydrates…

–IDI: Mother, 25 years, Westlands

Caregivers perceived food labels to be easy to read because in some cases they were presented in both English and Swahili and in simple language, but the font size of the labels are small. They made the following suggestions to improve on labelling: use of both Swahili and English on labels, and increasing the font size to make it easier to read and understand the information on labels. There was an emphasis on the use of simple English and translating the information on labels into other languages so that mothers and caregivers could read and understand it. They also pointed out the need for more explanations on the nutrition information provided and monitoring of processed and packaged foods to ensure safety.

…Their font size can be increased for them to be legible otherwise I'll continue ignoring them.

–IDI: Mother, 21 years, Westlands

…Okay, maybe they can try to put them in a language that is understandable to all like you know there are women who are not educated? Others can't just understand deep English. Some companies use really complex English which makes it hard to read.

–IDI: Mother, 25 years, Mathare

I would want the government to intervene and monitor the foods processed and packaged so they can make sure it is safe for the children including foods that are imported to avoid the sale of counterfeit products in the market.

–IDI: Mother, 28 years, Mathare

## 4 Discussion

This study aimed to understand caregiver perceptions and practices in relation to purchasing CPCFs. We found that consumption of CPCFs is relatively common among the study participants. The most consumed CPCF category was dry powdered and instant cereals and meals with chunky pieces. CPCFs were perceived to be healthy, nutritious, contain vitamins and minerals, cleaner and safer than non‐processed complementary foods, and clearly labelled by nearly all participants, a possible indication that the products are considered superior to non‐processed complementary foods. These perceptions varied by study setting, which is an indication that there is a need for contextualization of interventions around CPCFs. Other perceived benefits included prevention of childhood diseases, making children stronger, helping children gain weight, and keeping children active.

Consumption of CPCFs appears to be relatively high in urban areas in African countries (Debessa et al. 2023; Feeley et al. 2016) and other low and middle‐income countries (UNICEF East Asia and the Pacific Regional Office et al. 2023). In Ethiopia, for example, a study assessing the prevalence of the consumption of CPCFs among 386 caregivers with children aged 6‐23 months found that 44.5% of caregivers in the study offered their children CPCFs in the past 24 h of the reporting period (Debessa et al. 2023). The most common CPCFs offered were cereal‐based products. The study also reported that over half of the caregivers recruited in the study, 65.3% had a positive attitude towards CPCFs meaning that they thought CPCFs aid child growth, are convenient to use, and enhance a child's cognitive abilities. Similarly, in Senegal, Feeley et al. 2016 assessed the consumption of CPCFs as well as other products by children less than two years of age and the promotions of these products and found that 50% of caregivers offered their child a CPCF the most common being a cereal‐based product perceived to make babies smart. Our research findings show that food safety was an important consideration when purchasing CPCFs, which was based on perceptions around product packaging and ingredients used in the products. The quality of commercial complementary foods varies greatly, with some improving nutrient intake by providing micronutrients that may be lacking in young children's diets, while others are of concern due to particularly high levels of added salts, sugar or fats, or other unnecessary additives or ingredients (World Health Organization 2019).

Ease of preparation was considered a very important factor when purchasing CPCFs, which is a possible indication that convenience is key when it comes to the selection of complementary foods (Feeley et al. 2016). This can be explained by the fact that most mothers are in the workforce and therefore have limited time to allocate to childcare activities including food preparation (Debessa et al. 2023). To avoid overreliance on CPCFs which could potentially be harmful to child health, simple nutritious recipes on how to prepare complementary foods should be made available to mothers. Other factors that were very important included child preference, price, and taste. Similar findings have also been reported by other studies where child preference specifically is an important consideration when selecting CPCFs (Feeley 2016; Debessa et al. 2023). Concerns have been raised over the content and marketing of CPCFs, centring on the sweet‐taste profile of many foods; their lack of diverse ingredients (such as bitter vegetables); their nutritional quality (CPCFs are often lower in iron or higher in free sugars than homemade foods) and limited food textures. CPCFs are also criticized for lacking the authentic taste, texture and appearance of simple homemade foods (World Health Organization 2019; Cawley et al. 2015), which may negatively influence the acceptance of regular foods later in childhood. Regulations on the nutrient composition and marketing of these products could ensure that the products available in the market are age‐appropriate, healthy and safe for children. Caregivers should also be sensitized about CPCFs and their potential harm so that they are able to select healthy options for their children.

Health facilities were listed as the primary and trusted source of information on complementary feeding by a large proportion of study participants. This is encouraging as they are likely to get accurate information on infant feeding especially from professionals trained in Nutrition. However, it is worth noting that misleading information is sometimes provided by health workers, especially in cases where they are not adequately trained (Samuel et al. 2016). Health facilities were also listed as a source of information on CPCFs, but we did not explore the type of CPCFs that were being promoted within health facilities. Further exploration of this would enable us to establish if healthy or less healthy CPCFs are promoted and what further action needs to be taken to ensure that caregivers are adequately supported when making decisions about complementary feeding. Other sources of information on CPCFs included relatives and friends, which indicates the need to empower communities when it comes to infant feeding, as they have a strong influence on food choices by mothers (Karmacharya et al. 2017; Faye et al. 2019).

Media was also highlighted as a source of information on CPCFs, there is, therefore, a need to regulate media when it comes to the promotion of CPCFs to ensure that accurate information is relayed and healthy products are promoted. In Senegal, the most common source of information on CPCFs was television (40%) which claims that cereal‐based CPCFs make babies smart (Feeley 2016; Sy Gueye et al. 2016). The use of media such as TV appears to be effective in influencing maternal perceptions as making the baby smart was one of the reasons caregivers opted to purchase cereal‐based CPCFs in Dakar (Feeley 2016). Marketing through social media is also common, but we did not capture this information.

CPCFs were also marketed in stores, and this caught the attention of the caregivers. Marketing strategies used in stores included price reductions and gifts while strategies used on product packaging included the use of words such as kiddos, toto, and baby weaning. The age of the target group was also indicated on some of the products. Nutrition and health claims were also included in product packaging, which can be misleading especially if the products do not contain the listed nutrients. Similar findings have previously been reported (García et al. 2019)

Inappropriate promotion of commercial complementary foods and beverages can mislead and confuse caregivers about the nutrition and health‐related qualities of these foods and beverages and about their age‐appropriate and safe use (World Health Organization 2017). The use of Nutrient Profiling Models (NPMs) for complementary foods could benefit Kenya as it would enable the classification of CPCFs as healthy or less healthy and in turn regulate their promotion and marketing. The creation of such a model could build on the KNPM that is currently being finalized and aims to control excessive consumption of nutrients of public health concern towards prevention and control of diet‐related NCDs among adults.

Regulations on the use of health and nutrition claims may be beneficial to caregivers in the two settings as they appear to rely on this information to select CPCFs. This can be implemented together with other strategies such as nutrient profiling, training, information provision and or withdrawal and education (Shroff et al. 2012). These, coupled with improved labelling may also go a long way to ensure that caregivers are able to understand the information presented on the product and in turn make informed decisions (Cawley et al. 2015; Feunekes et al. 2008).

This study enabled us to document perceptions on CPCFs among caregivers in one urban slum and one non‐slum setting. CPCFs were also identified, and marketing strategies on product packaging were documented amidst some challenges and limitations, which included limited access to larger supermarkets because of refusal from the target supermarkets to participate in the study. This meant that not all CPCFs available in the market were captured. More engagement with supermarket owners is required to ensure their cooperation in such studies, given that they are a major stakeholder. In Mathare, we also focused on supermarkets, which means that we may not have captured all the products that are sold in smaller establishments such as kiosks and markets. The study included a limited sample of participants primarily because it was a pilot study; hence, the findings from this study are not generalizable. A larger study that includes caregivers in rural settings and health workers, given their role in relaying information on CPCFs is warranted. The use of health and nutrition claims on product packaging needs to be further explored. There is potential to analyse the food products to assess whether the claims made are valid. The differences in perceptions between slum and non‐slum residents warrant contextualization of interventions related to CPCFs to effectively address the needs of caregivers in each setting.

We found that CPCFs are highly regarded by caregivers and are commonly used in slum (Mathare) and non‐slum (Westlands) settings in Nairobi. This could potentially have a negative impact on child health if their composition and marketing/promotion is not regulated given that health and nutrition claims are commonly used to market them. More research on the composition of these products is required. Regulation on the promotion and marketing of CPCFs is also required and Kenya could potentially benefit from a Nutrient Profiling Model targeting complementary foods.

## Author Contributions

Antonina N. Mutoro, Elizabeth Kimani‐Murage, Charity Zvandaziva, and Gershim Asiki designed the study. Antonina N. Mutoro, Maureen Gitagia, and Veronica Sanda Ojiambo performed the research. Antonina N. Mutoro and Maureen Gitagia drafted the manuscript. All authors reviewed the manuscript and approved the final version.

## Conflicts of Interest

The authors declare no conflicts of interest.

## Supporting information

Supplementary_material_observation_guide.

Supplementary_Material_Qualitative_Tool.

Supplementary_Material_Interview_Guide.

Supplementary_tables_and_figures.

## Data Availability

The data that support the findings of this study are available from the corresponding author upon reasonable request.
