# Supplementary material for: Understanding the Purchasing and Consumption Dynamics of Commercially Processed Complementary Foods and Caregiver Motivations and Reasons for Purchasing These Foods in Nairobi
Source: Matern Child Nutr. 2025 Sep 7;22(1):e70102. doi: 10.1111/mcn.70102 (PMC12893508; doi:10.1111/mcn.70102)
Supplement: Supplementary file 1 — Supplementary_material_observation_guide. [file MCN-22-e70102-s003.pdf]

In Store Observations

Field	Question	Answer
intronote	African Population and Health Research Center<br>UNICEF Project<br>In store Observations<br>© APHRC 2023	
consent_note	**CONSENT NOTE:**	
consent *(required)*	**ENUMERATOR NOTE:**<br>Has the respondent consented to be interviewed?	1 Yes<br>0 No
consent_given_grp<br>*Group relevant when: selected(${consent} ,'1')*		
enumerator *(required)*	**PLEASE SELECT THE FIELD INTERVIEWER'S CODE:**	Lucas_Muturi Lucas Muturi<br>Linus_Kamande Linus Kamande<br>Jackline_Nduta Jackline Nduta<br>Mercy_Obim Mercy Obim<br>Sophia_Wairimu Sophia Wairimu<br>Hannah_Nyawira Hannah Nyawira<br>Eunice_Nyambura Eunice Nyambura<br>Grace_Ndegwa Grace Ndegwa
interview_date *(required)*	**DATE OF INTERVIEW:**<br>*(DD/MM/YYYY)*<br>*Response constrained to: .<=today()*	
consent_given_grp > store_details_grp		
store_details_grp_label	**Store Details**	
location *(required)*	**Location:**	1 Mathare<br>2 Westlands
store_name *(required)*	**Store Name:**	1 Mathare<br>2 Royal Mart<br>3 Lango Supermarket<br>4 Hope Supermarket<br>5 Confidence Supermarket<br>6 KANGEMI SUPERMARKET<br>7 MOUNTAIN MINI MART<br>8 RUNDA MINI MART<br>9 SERABEN SUPERMARKET<br>10 Quickmatt<br>11 Chandarana<br>12 Fair value<br>13 Mantex<br>14 Nitt<br>15 Mountain Mini-matt<br>16 Pamoja supermarket<br>17 Friendly 5<br>18 Friends Supermarket

Field	Question	Answer
store_type *(required)*	**Store Type:**	1 Major supermarket<br>2 Mini supermarket<br>96 Other (Specify)
store_type_specify *(required)*	**Specify other Store Type**<br>*Question relevant when: selected(${store_type} ,'96')*	
consent_given_grp > cpcfs_grp		
cpcfs_grp_label	**Types of CPCFs sold/advertised in the outlet**<br>Indicate whether the listed commercially processed complementary foods in the table below are sold and or advertised in store	
cpcfs_beverage_label	**Juices and other beverages**	1 Yes<br>0 No
cpcfs_beverage_sold *(required)*	Sold	1 Yes<br>0 No
cpcfs_beverage_advert *(required)*	Advertised	1 Yes<br>0 No
cpcfs_starch_label	**Dry, powdered and instant cereal/starchy food**	1 Yes<br>0 No
cpcfs_starch_sold *(required)*	Sold	1 Yes<br>0 No
cpcfs_starch_advert *(required)*	Advertised	1 Yes<br>0 No
cpcfs_ready_to_eat_label	**Soft–wet spoon-able, ready-to-eat foods, typically smooth or semi-pureed packaged in jars or pouches and can be spoon-fed (purees)**	1 Yes<br>0 No
cpcfs_ready_to_eat_sold *(required)*	Sold	1 Yes<br>0 No
cpcfs_ready_to_eat_advert *(required)*	Advertised	1 Yes<br>0 No
cpcfs_meals_label	**Meals with chunky pieces, often sold in trays or pots for older infants and young children**	1 Yes<br>0 No
cpcfs_meals_sold *(required)*	Sold	1 Yes<br>0 No
cpcfs_meals_advert *(required)*	Advertised	1 Yes<br>0 No
cpcfs_snacks_label	**Dry finger foods and snacks**	1 Yes<br>0 No
cpcfs_snacks_sold *(required)*	Sold	1 Yes<br>0 No
cpcfs_snacks_advert *(required)*	Advertised	1 Yes<br>0 No
consent_given_grp > strategy_grp<br>*Group relevant when: selected(${calc_ask_marketing} ,'1')*		
strategy_grp_label	**Strategies used to advertise/ promote various food items**<br><br>**ENUMERATOR NOTE:**<br>Select all the strategies used for each food item	
strategy_beverage_advert *(required)*	**Strategies used for:**<br><br>**Juices and other beverages**<br>*Select all that apply*<br>*Question relevant when: selected(${cpcfs_beverage_advert} ,'1')*<br>*Response constrained to: not(selected(., '98') and count-selected(.) > 1)*	1 Entertainers/animators<br>2 Magazine of discounts when entering the store<br>3 Discount coupons for activities (e.g., cinema)<br>4 Price reductions<br>5 Price comparisons with other stores<br>6 Discount (%, 3x2, among others)<br>7 Additional gift (toys, utensils, among others)<br>8 Promotion on packaging (characters, cartoons, celebrities, athletes, events, among others...)<br>9 Promoters (with tasting)

Field	Question	Answer
		10 Promoters (without tasting)<br>11 Special exhibitions on the shelf<br>12 Block display<br>98 Not applicable<br>96 Other (Specify)
strategy_beverage_advert_specify *(required)*	Other (Specify)<br>*Question relevant when: selected(${strategy_beverage_advert} ,'96')*	
strategy_starch_advert *(required)*	**Strategies used for:**<br><br>**Dry, powdered and instant cereal/starchy food**<br>*Select all that apply*<br>*Question relevant when: selected(${cpcfs_starch_advert} ,'1')*<br>*Response constrained to: not(selected(., '98') and count-selected(.) > 1)*	1 Entertainers/animators<br>2 Magazine of discounts when entering the store<br>3 Discount coupons for activities (e.g., cinema)<br>4 Price reductions<br>5 Price comparisons with other stores<br>6 Discount (%, 3x2, among others)<br>7 Additional gift (toys, utensils, among others)<br>8 Promotion on packaging (characters, cartoons, celebrities, athletes, events, among others...)<br>9 Promoters (with tasting)<br>10 Promoters (without tasting)<br>11 Special exhibitions on the shelf<br>12 Block display<br>98 Not applicable<br>96 Other (Specify)
strategy_starch_advert_specify *(required)*	Other (Specify)<br>*Question relevant when: selected(${strategy_starch_advert} ,'96')*	
strategy_ready_to_eat_advert *(required)*	**Strategies used for:**<br><br>**Soft–wet spoon-able, ready-to-eat foods, typically smooth or semi-pureed packaged in jars or pouches and can be spoon-fed (purees)**<br>*Select all that apply*<br>*Question relevant when: selected(${cpcfs_ready_to_eat_advert} ,'1')*<br>*Response constrained to: not(selected(., '98') and count-selected(.) > 1)*	1 Entertainers/animators<br>2 Magazine of discounts when entering the store<br>3 Discount coupons for activities (e.g., cinema)<br>4 Price reductions<br>5 Price comparisons with other stores<br>6 Discount (%, 3x2, among others)<br>7 Additional gift (toys, utensils, among others)<br>8 Promotion on packaging (characters, cartoons, celebrities, athletes, events, among others...)<br>9 Promoters (with tasting)<br>10 Promoters (without tasting)<br>11 Special exhibitions on the shelf<br>12 Block display<br>98 Not applicable<br>96 Other (Specify)
strategy_ready_to_eat_advert_specify *(required)*	Other (Specify)<br>*Question relevant when: selected(${strategy_ready_to_eat_advert} ,'96')*	
strategy_meals_advert *(required)*	**Strategies used for:**<br><br>**Meals with chunky pieces, often sold in trays or pots for older infants and young children**	1 Entertainers/animators<br>2 Magazine of discounts when entering the store

Field	Question	Answer
	Select all that apply<br>*Question relevant when: selected(${cpcfs_meals_advert} ,'1')*<br>*Response constrained to: not(selected(., '98') and count-selected(.) > 1)*	3 Discount coupons for activities (e.g., cinema)<br>4 Price reductions<br>5 Price comparisons with other stores<br>6 Discount (%, 3x2, among others)<br>7 Additional gift (toys, utensils, among others)<br>8 Promotion on packaging (characters, cartoons, celebrities, athletes, events, among others...)<br>9 Promoters (with tasting)<br>10 Promoters (without tasting)<br>11 Special exhibitions on the shelf<br>12 Block display<br>98 Not applicable<br>96 Other (Specify)
strategy_meals_advert_specify *(required)*	Other (Specify)<br>*Question relevant when: selected(${strategy_meals_advert} ,'96')*	
strategy_snacks_advert *(required)*	**Strategies used for:**<br><br>**Dry finger foods and snacks**<br>*Select all that apply*<br>*Question relevant when: selected(${cpcfs_snacks_advert} ,'1')*<br>*Response constrained to: not(selected(., '98') and count-selected(.) > 1)*	1 Entertainers/animators<br>2 Magazine of discounts when entering the store<br>3 Discount coupons for activities (e.g., cinema)<br>4 Price reductions<br>5 Price comparisons with other stores<br>6 Discount (%, 3x2, among others)<br>7 Additional gift (toys, utensils, among others)<br>8 Promotion on packaging (characters, cartoons, celebrities, athletes, events, among others...)<br>9 Promoters (with tasting)<br>10 Promoters (without tasting)<br>11 Special exhibitions on the shelf<br>12 Block display<br>98 Not applicable<br>96 Other (Specify)
strategy_snacks_advert_specify *(required)*	Other (Specify)<br>*Question relevant when: selected(${strategy_snacks_advert} ,'96')*	
informas_repeat_label	**INFORMAS Tool**<br><br>The following sections of the INFORMAS tool will be used to collect information from commercially processed complementary foods	
consent_given_grp > Informas (1)		(Repeated group)
consent_given_grp > Informas (1) > informas_sub_grp_1		
food_type *(required)*	Type of food	
classification *(required)*	Classification	1 Juices and other beverages<br>2 Dry, powdered and instant cereal/starchy food<br>3 Soft–wet spoon-able, ready-to-eat foods, typically smooth or semi-pureed packaged in jars or

Field	Question	Answer
		pouches and can be spoon-fed (purees)<br>4 Meals with chunky pieces, often sold in trays or pots for older infants and young children,<br>5 Dry finger foods and snacks
ingredients *(required)*	Ingredient list	1 Yes<br>0 No
nutrients *(required)*	Nutrient declarations	1 Yes<br>0 No
consent_given_grp > Informas (1) > nutrients_grp<br>*Group relevant when: selected(${nutrients} ,'1')*		
nutrients_grp_label	**Nutrition composition (amounts)**	
free_sugars *(required)*	Free Sugars (g)<br>*[Indicate -98 if "Don't Know"]*<br>*Response constrained to: .>=0 or .=-98*	
salt_sodium *(required)*	Salt/ Sodium (mg)<br>*[Indicate -98 if "Don't Know"]*<br>*Response constrained to: .>=0 or .=-98*	
saturated_fats *(required)*	Saturated Fats (g)<br>*[Indicate -98 if "Don't Know"]*<br>*Response constrained to: .>=0 or .=-98*	
trans_fats *(required)*	Trans Fats (g)<br>*[Indicate -98 if "Don't Know"]*<br>*Response constrained to: .>=0 or .=-98*	
fats *(required)*	Fats (g)<br>*[Indicate -98 if "Don't Know"]*<br>*Response constrained to: .>=0 or .=-98*	
consent_given_grp > Informas (1) > informas_sub_grp_2		
nutrition_claim *(required)*	Nutrition on claim	1 Yes<br>0 No
nutrition_location *(required)*	Nutrition claim location on package<br>*Question relevant when: selected(${nutrition_claim} ,'1')*	1 Front side<br>2 Back side<br>3 sides
nutrition_location_word *(required)*	Wording used (specific claim)<br>*Question relevant when: selected(${nutrition_claim} ,'1')*	
health_claim *(required)*	Health claim	1 Yes<br>0 No
health_location *(required)*	Health claim location on package<br>*Question relevant when: selected(${health_claim} ,'1')*	1 Front side<br>2 Back side<br>3 sides
health_location_word *(required)*	Wording used (specific claim)<br>*Question relevant when: selected(${health_claim} ,'1')*	
consent_given_grp > Informas (1) > promotions_grp		
promotions_grp_label	**Promotion tactics**	1 Yes<br>0 No
cartoons *(required)*	Cartoons	1 Yes<br>0 No
free_samples *(required)*	Free samples	1 Yes<br>0 No
games *(required)*	Games	1 Yes<br>0 No
toys *(required)*	Toys	1 Yes<br>0 No
images_of_children *(required)*	Images of children	1 Yes<br>0 No
thank_you_note	**ENUMERATOR NOTE:**<br><br>**THANK THE RESPONDENT FOR PARTICIPATION**	
ENDING THE INTERVIEW		
gps_coordinates	**GPS coordinates**	

Field	Question	Answer
	ENUMERATOR NOTE:<br><br>GPS coordinates can only be captured accurately when outside the building.<br>*GPS coordinates can only be collected when outside.*	
general_comments	RECORD ANY GENERAL COMMENTS	
