# Supplementary material for: Understanding the Purchasing and Consumption Dynamics of Commercially Processed Complementary Foods and Caregiver Motivations and Reasons for Purchasing These Foods in Nairobi
Source: Matern Child Nutr. 2025 Sep 7;22(1):e70102. doi: 10.1111/mcn.70102 (PMC12893508; doi:10.1111/mcn.70102)
Supplement: Supplementary file 2 — Supplementary_Material_Qualitative_Tool. [file MCN-22-e70102-s001.docx]

**Qualitative Tool**

**Understanding the purchasing and consumption dynamics of commercially processed complementary foods and caregiver motivations and reasons for purchasing these foods in Kenya**

1. What do you generally think about Commercially Processed Complementary Foods?

*Probe: what do you think about their health benefits// nutrition quality for children?*

*Probe: What do you think about the safety of CPCFs for children?*

1. What made you start purchasing CPCFs food?
2. What do you look out for when buying CPCFs?

*Probe: Do you consider food labels? The manufacturer? The packaging? when making purchase decisions*

*● Why do you read food labels and do you think they are important?*

*● In your view, are the labels easy or hard to read and understand?*

*● What can be done to make labels easy to understand?*

1. How do you use the information presented on the labels?
    *Probe: How does this information help you decide the type of food to buy?*
