# Supplementary material for: Understanding the Purchasing and Consumption Dynamics of Commercially Processed Complementary Foods and Caregiver Motivations and Reasons for Purchasing These Foods in Nairobi
Source: Matern Child Nutr. 2025 Sep 7;22(1):e70102. doi: 10.1111/mcn.70102 (PMC12893508; doi:10.1111/mcn.70102)
Supplement: Supplementary file 3 — Supplementary_Material_Interview_Guide. [file MCN-22-e70102-s004.pdf]

Field	Question	Answer
		15 Mountain Mini-matt<br>16 Pamoja supermarket<br>17 Friendly 5<br>18 Friends Supermarket
store_type *(required)*	**Store Type:**	1 Large Supermarket<br>2 Medium supermarket<br>96 Other (Specify)
store_type_specify *(required)*	**Specify other Store Type**<br>*Question relevant when: selected(${store_type} ,'96')*	
consent_given_grp > caregiver_grp		
caregiver_grp_label	**Section 2: Caregivers Information**	
consent_given_grp > caregiver_grp > caregiver_sub_grp_1		
q2_1 *(required)*	**2.1** Gender<br>*Jinsia*	1 Male<br>2 Female
q2_2 *(required)*	**2.2.** How old are you?<br>*[Indicate 998 for "Don't Know"*<br>*Response constrained to: (.>=15 and .<=130) or .=998*	
q2_3 *(required)*	**2.3.** When was your child born?<br>*(DD/MM/YYYY)*<br>*Response constrained to: .<today()*	
q2_4 *(required)*	**2.4.** What is your religion?	1 Christian<br>2 Muslim<br>3 Traditional<br>4 Hindu<br>5 Not willing to answer<br>96 Other (Specify)
q2_4_specify *(required)*	Specify other religion<br>*Question relevant when: selected(${q2_4} ,'96')*	
q2_5 *(required)*	**2.5.** What is the highest level of education that you have completed?	1 No formal schooling<br>2 Pre-school<br>3 Primary school<br>4 Secondary school<br>5 College / University<br>6 Vocational Training<br>99 Not willing to answer
q2_6 *(required)*	**2.6.** What is your marital status?	1 Currently married<br>2 Living together<br>3 Separated<br>4 Divorced<br>5 Widowed<br>6 Never married<br>99 Not willing to answer
q2_6a *(required)*	**2.6a.** What is the highest level of education that your **spouse completed**?<br>*Question relevant when: selected(${q2_6} ,'1') or selected(${q2_6} ,'2')*	1 No formal schooling<br>2 Pre-school<br>3 Primary school<br>4 Secondary school<br>5 College / University<br>6 Vocational Training<br>7 Not willing to answer<br>98 Don't Know
q2_7 *(required)*	**2.7.** Source of income	1 Employee<br>2 Entrepreneur / self-employed /worker in your own household<br>3 Unemployed<br>4 Casual Labour<br>96 Other (Specify)<br>99 Not willing to answer
q2_7_specify *(required)*	Specify other main source of livelihood<br>*Question relevant when: selected(${q2_7} ,'96')*	
q2_8 *(required)*	**2.8.** What is your household's monthly income?	1 <10,000<br>2 10,001-20,000<br>3 20,001-30,000

[illegible]

Field	Question	Answer
	**The reason I buy CPCFs is…**	4 Agree<br>5 Totally agree<br>6 I don't Know<br>7 Not willing to answer
q3_6a *(required)*	They are healthy	1 Totally disagree<br>2 Disagree<br>3 Neutral<br>4 Agree<br>5 Totally agree<br>6 I don't Know<br>7 Not willing to answer
q3_6b *(required)*	They are nutritious	1 Totally disagree<br>2 Disagree<br>3 Neutral<br>4 Agree<br>5 Totally agree<br>6 I don't Know<br>7 Not willing to answer
q3_6c *(required)*	They contain a lot of vitamins and minerals	1 Totally disagree<br>2 Disagree<br>3 Neutral<br>4 Agree<br>5 Totally agree<br>6 I don't Know<br>7 Not willing to answer
q3_6d *(required)*	They are high in protein	1 Totally disagree<br>2 Disagree<br>3 Neutral<br>4 Agree<br>5 Totally agree<br>6 I don't Know<br>7 Not willing to answer
q3_6e *(required)*	They are cleaner and safer than non-processed complementary foods repared meals	1 Totally disagree<br>2 Disagree<br>3 Neutral<br>4 Agree<br>5 Totally agree<br>6 I don't Know<br>7 Not willing to answer
q3_6f *(required)*	They are easy to prepare	1 Totally disagree<br>2 Disagree<br>3 Neutral<br>4 Agree<br>5 Totally agree<br>6 I don't Know<br>7 Not willing to answer
q3_6g *(required)*	They are cheap	1 Totally disagree<br>2 Disagree<br>3 Neutral<br>4 Agree<br>5 Totally agree<br>6 I don't Know<br>7 Not willing to answer
q3_6h *(required)*	My child prefers them to foods prepared at home	1 Totally disagree<br>2 Disagree<br>3 Neutral<br>4 Agree<br>5 Totally agree<br>6 I don't Know<br>7 Not willing to answer

Field	Question	Answer
q3_6i *(required)*	They are popular	1 Totally disagree<br>2 Disagree<br>3 Neutral<br>4 Agree<br>5 Totally agree<br>6 I don't Know<br>7 Not willing to answer
q3_6j *(required)*	They are clearly labelled and provide information about nutrition and health	1 Totally disagree<br>2 Disagree<br>3 Neutral<br>4 Agree<br>5 Totally agree<br>6 I don't Know<br>7 Not willing to answer
consent_given_grp > purchase_grp > q3_1_grp > q3_7_grp		
q3_7_grp_label	**3.7.** How important are the following factors to you when you decide to buy CPCFs?	1 Totally unimportant<br>2 Unimportant<br>3 Neutral<br>4 Important<br>5 Absolutely important
q3_7a *(required)*	Taste	1 Totally unimportant<br>2 Unimportant<br>3 Neutral<br>4 Important<br>5 Absolutely important
q3_7b *(required)*	Personal Preference	1 Totally unimportant<br>2 Unimportant<br>3 Neutral<br>4 Important<br>5 Absolutely important
q3_7c *(required)*	Food safety	1 Totally unimportant<br>2 Unimportant<br>3 Neutral<br>4 Important<br>5 Absolutely important
q3_7d *(required)*	Nutrition quality	1 Totally unimportant<br>2 Unimportant<br>3 Neutral<br>4 Important<br>5 Absolutely important
q3_7e *(required)*	Value for money	1 Totally unimportant<br>2 Unimportant<br>3 Neutral<br>4 Important<br>5 Absolutely important
q3_7f	Easily available	1 Totally unimportant<br>2 Unimportant<br>3 Neutral<br>4 Important<br>5 Absolutely important
q3_7g *(required)*	Ease of preparing	1 Totally unimportant<br>2 Unimportant<br>3 Neutral<br>4 Important<br>5 Absolutely important
q3_7h *(required)*	Labelling	1 Totally unimportant<br>2 Unimportant<br>3 Neutral<br>4 Important<br>5 Absolutely important

Field	Question	Answer
q3_7i *(required)*	Price	1 Totally unimportant<br>2 Unimportant<br>3 Neutral<br>4 Important<br>5 Absolutely important
q3_7j *(required)*	Child preference	1 Totally unimportant<br>2 Unimportant<br>3 Neutral<br>4 Important<br>5 Absolutely important
consent_given_grp > iycf_src_grp		
iycf_src_grp_label	**Section 4: Sources of information on infant and young child feeding/CPCFs**	
q4_1 *(required)*	**4.1.** Have you ever received any information on complementary feeding practices?	1 Yes<br>0 No
consent_given_grp > iycf_src_grp > q4_1_grp<br>*Group relevant when: selected(${q4_1} ,'1')*		
q4_2 *(required)*	**4.2.** Where did you receive the information about complementary feeding from?<br>*[MULTIPLE RESPONSES ALLOWED]*	1 Antenatal /MCH Clinics /Health Facility<br>2 Community Health Volunteers<br>3 Relative/Friend/Neighbour<br>4 Community Barraza<br>5 Mother to Mother Support Group<br>6 Information Education and Communication Material (IEC)/Print Media<br>7 Media (Radio/TV)<br>8 Traditional Birth Attendant<br>9 Religious Places (Church's, Mosque)<br>10 NGO/CBOs<br>11 Outreach Services/Campaigns<br>12 Day Care Centre/Early Childhood Development center (ECD)<br>13 Chama<br>96 Other (Specify)
q4_2_specify *(required)*	Specify other<br>*Question relevant when: selected(${q4_2} ,'96')*	
q4_3	**4.3.** Which of these sources is the MOST trusted?	1 Antenatal /MCH Clinics /Health Facility<br>2 Community Health Volunteers<br>3 Relative/Friend/Neighbour<br>4 Community Barraza<br>5 Mother to Mother Support Group<br>6 Information Education and Communication Material (IEC)/Print Media<br>7 Media (Radio/TV)<br>8 Traditional Birth Attendant<br>9 Religious Places (Church's, Mosque)<br>10 NGO/CBOs<br>11 Outreach Services/Campaigns<br>12 Day Care Centre/Early Childhood Development center (ECD)<br>13 Chama<br>96 Other (Specify)
q4_3_specify *(required)*	Specify other<br>*Question relevant when: selected(${q4_3} ,'96')*	

Field	Question	Answer
q4_4 *(required)*	**4.4.** Have you ever received any information on commercially processed complementary foods?	1 Yes<br>0 No
consent_given_grp > iycf_src_grp > q4_4_grp<br>*Group relevant when: selected(${q4_4} ,'1')*		
q4_5 *(required)*	**4.5.** Where did you receive the information about commercially processed complementary foods (CPCFs) from?<br>*[MULTIPLE RESPONSES ALLOWED]*	1 Antenatal /MCH Clinics /Health Facility<br>2 Community Health Volunteers<br>3 Relative/Friend/Neighbour<br>4 Community Barraza<br>5 Mother to Mother Support Group<br>6 Information Education and Communication Material (IEC)/Print Media<br>7 Media (Radio/TV)<br>8 Traditional Birth Attendant<br>9 Religious Places (Church's, Mosque)<br>10 NGO/CBOs<br>11 Outreach Services/Campaigns<br>12 Day Care Centre/Early Childhood Development center (ECD)<br>13 In-store advertisements<br>14 Chama<br>96 Other (Specify)
q4_5_specify *(required)*	Specify other<br>*Question relevant when: selected(${q4_5} ,'96')*	
q4_6 *(required)*	**4.6.** Did you hear or see an advert for commercially processed complementary foods in the shop when you were shopping	1 Yes<br>0 No
q4_7 *(required)*	**4.7.** Can you tell me about some of the things you saw or heard?<br>*[MULTIPLE RESPONSES ALLOWED]*<br>*Question relevant when: selected(${q4_6} ,'1')*	1 Entertainers / animators<br>2 Magazine of discounts when entering the store<br>3 Discount coupons for activities (e.g cinema)<br>4 Price reductions<br>5 Price comparison with other stores<br>6 Additional gifts<br>7 Promotion on packaging (characters, cartoons, celebrities,<br>8 Special exhibitions on the shelf<br>9 Book display<br>96 Other (Specify)
q4_7_specify *(required)*	Specify other<br>*Question relevant when: selected(${q4_7} ,'96')*	
consent_given_grp > marketing_grp		
marketing_grp_label	**Section 5: In store marketing**	
q5_1 *(required)*	**5.1.** Did you buy any item that you saw/heard in an advertisement in the shop?	1 Yes<br>0 No
consent_given_grp > marketing_grp > q5_1_yes_grp<br>*Group relevant when: selected(${q5_1} ,'1')*		
q5_2 *(required)*	**5.2.** Will you say you bought those items in response to the adverts or promotions you saw or heard in the shop?	1 Yes<br>0 No
q5_3 *(required)*	**5.3.** Before buying a product, do you read or look at the front of package information displayed on the product?	1 Yes<br>0 No
q5_4 *(required)*	**5.4.** The items you bought, in terms of shelf placement, where were they placed?	1 Top shelf<br>2 Eye my level<br>3 Bottom of the shelf

Field	Question	Answer
q5_5 *(required)*	**5.5.** The items you bought, in terms of shop placement, where were they placed?	1 Prominent (eye level placement)<br>2 Noticeable (when I go looking I see the product)<br>3 In conspicuous (after I have been looking for a while, I see the product)
consent_given_grp > marketing_grp > q5_6_grp		
q5_6_grp_label	**5.6.** Please mark whether you agree or disagree with the following statements for the store where you buy most of your food and your shopping habits at that store.	1 Totally disagree<br>2 Disagree<br>3 Neutral<br>4 Agree<br>5 Totally agree<br>6 I don't Know<br>7 Not willing to answer
q5_6a *(required)*	I notice signs that encourage me to purchase commercially processed foods	1 Totally disagree<br>2 Disagree<br>3 Neutral<br>4 Agree<br>5 Totally agree<br>6 I don't Know<br>7 Not willing to answer
q5_6b *(required)*	I see nutrition labels or nutrition information for most packaged CPCFs at the stores	1 Totally disagree<br>2 Disagree<br>3 Neutral<br>4 Agree<br>5 Totally agree<br>6 I don't Know<br>7 Not willing to answer
thank_you_note	**ENUMERATOR NOTE:**<br><br>**THANK THE RESPONDENT FOR PARTICIPATION**	
ENDING THE INTERVIEW		
gps_coordinates	**GPS coordinates**<br><br>**ENUMERATOR NOTE:**<br><br>GPS coordinates can only be captured accurately when outside the building.<br>*GPS coordinates can only be collected when outside.*	
general_comments	RECORD ANY GENERAL COMMENTS	
