# Supplementary material for: Understanding the Purchasing and Consumption Dynamics of Commercially Processed Complementary Foods and Caregiver Motivations and Reasons for Purchasing These Foods in Nairobi
Source: Matern Child Nutr. 2025 Sep 7;22(1):e70102. doi: 10.1111/mcn.70102 (PMC12893508; doi:10.1111/mcn.70102)
Supplement: Supplementary file 4 — Supplementary_tables_and_figures. [file MCN-22-e70102-s002.docx]

***Understanding the purchasing and consumption dynamics of commercially processed complementary foods and caregiver motivations and reasons for purchasing these foods in Nairobi.***

*Supplementary Table 1: A summary of study methods by objective*

| **Study objective** | **Methods/study sites** | **Outcomes/themes** | **Sample size and sampling** |
| --- | --- | --- | --- |
| 1. Assess the purchasing and consumption dynamics of CPCF | Quantitative cross sectional survey study-exit interviews of caregivers in retail food outlets (Nairobi) | frequency, place of purchases, types of products purchased, and use of products | Sample size: 80 caregivers.  Purposive and stratified sampling: 2 strata  Nairobi-urban slum  Nairobi-non-slum (figure 1) |
| 2. Map and describe sources of information for caregivers on infant and young child feeding | Quantitative cross-sectional survey as in with the same participants as in 1 | Sources of information from respondents | Same as in 1 |
| 3. Explore caregiver motivations and reasons for purchasing CPCFs | Qualitative interviews (In depth interviews s) with caregivers | Motivations and reasons for purchasing CPCFs | 16 interviews (8 from each stratum |
| 4.Understand caregivers’ perception of nutrition and health claims and other labels and how this influences their purchasing decisions | Qualitative interviews with same respondents as in 3 | Perceptions  Influence on decisions | Interviews with the same caregivers in 3 |
| b. To document types of marketing strategies used in stores and on product packaging of CPCFs | In-store observations using INFORMAS checklists for marketing | Marketing information in stores and strategies used | 8 retail outlets |
| Stakeholder engagement | Desk review of Kenyan NPM  Consultative meetings | Identification of gaps in the current NPM | Ministry of Health task force working with other stakeholders |

*Supplementary Table 2: Reasons why caregivers purchase CPCFs n=81*

| **Reason** | **Total (%)** | **Mathare (%)** | **Westlands (%)** | **P value** |
| --- | --- | --- | --- | --- |
| **They are healthy (n=78)** |  |  |  |  |
| Disagree | 1.3 |  | 2.6 | 0.302 |
| Agree | 98.7 | 100 | 97.4 |  |
| **They are nutritious (n=78)** |  |  |  |  |
| Disagree | 2.6 | 0 | 5.3 | 0.215 |
| Neutral | 1.3 | 2.5 | 0 |  |
| Agree | 96.2 | 97.5 | 94.7 |  |
| **They contain a lot of vitamins and minerals (n=77)** |  |  |  |  |
| Disagree | 5.2 | 5.0 | 5.4 | 0.931 |
| Neutral | 6.5 | 7.5 | 5.4 |  |
| Agree | 88.3 | 87.5 | 89.2 |  |
| **They are high in protein (n=76)** |  |  |  |  |
| Disagree | 7.9 | 10.0 | 5.6 | 0.770 |
| Neutral | 10.5 | 10.0 | 11.1 |  |
| Agree | 81.6 | 80.0 | 83.3 |  |
| **They are cleaner and safer than non-processed complementary foods prepared meals (n=77)** |  |  |  |  |
| Disagree | 10.4 | 0 | 21.6 | 0.008 |
| Neutral | 2.6 | 2.5 | 2.7 |  |
| Agree | 87.0 | 97.5 | 75.7 |  |
| **They are easy to prepare (n=75)** |  |  |  |  |
| Disagree | 4.0 | 2.6 | 5.6 | 0.801 |
| Neutral | 2.7 | 2.6 | 2.8 |  |
| Agree | 93.3 | 94.8 | 91.6) |  |
| **They are cheap (n=78)** |  |  |  |  |
| Disagree | 43.6 | 45.0 | 42.1 | 0.560 |
| Neutral | 14.1 | 17.5 | 10.5 |  |
| Agree | 42.3 | 37.5 | 47.4 |  |
| **My child prefers them to foods prepared at home (n=77)** |  |  |  |  |
| Disagree | 19.5 | 23.1 | 15.8 | 0.148 |
| Neutral | 14.3 | 20.5 | 7.9 |  |
| Agree | 66.2 | 56.4 | 76.3 |  |
| **They are popular (n=78)** |  |  |  |  |
| Disagree | 20.5 | 15.4 | 25.6 | 0.223 |
| Neutral | 10.3 | 15.4 | 5.1 |  |
| Agree | 69.2 | 69.2 | 69.2 |  |
| **They are clearly labeled and provide information about nutrition and health (n=77)** |  |  |  |  |
| Disagree | 7.8 | 0 | 15.8 | 0.023 |
| Neutral | 1.3 | 2.6 | 0 |  |
| Agree | 90.9 | 97.4 | 84.4 |  |

*Supplementary Table 3: Factors that are considered when purchasing CPCFs n=81*

| Reason | Total | Mathare (n%) | Westlands (n%) | P value |
| --- | --- | --- | --- | --- |
| **Taste** |  |  |  |  |
| Unimportant | 1.2 | 0 | 2.4 | 0.368 |
| Neutral | 1.2 | 2.5 | 0 |  |
| Important | 97.6 | 97.5 | 97.6 |  |
| **Personal preference** |  |  |  |  |
| Unimportant | 6.2 | 10.0 | 2.4 | 0.310 |
| Neutral | 6.2 | 7.5 | 4.9 |  |
| Important | 87.6 | 82.5 | 92.7 |  |
| **Food safety** |  |  |  |  |
| Important | 100 | 100 | 100 |  |
| **Nutrition quality** |  |  |  |  |
| Unimportant | 2.5 | 0 | 4.9 | 0.368 |
| Neutral | 2.5 | 2.5 | 2.4 |  |
| Important | 95.0 | 97.5 | 92.7 |  |
| **Value for money** |  |  |  |  |
| Unimportant | 4.9 | 2.5 | 7.3 | 0.241 |
| Neutral | 6.2 | 10.0 | 2.4 |  |
| Important | 88.9 | 87.5 | 90.2 |  |
| **Easily available n=75** |  |  |  |  |
| Unimportant | 4.0 | 7.7 | 0 | 0.001 |
| Neutral | 12.0 | 23.1 | 0 |  |
| Important | 84.0 | 69.2 | 100 |  |
| **Ease of preparing** |  |  |  |  |
| Unimportant | 2.5 | 5.0 | 0 | 0.349 |
| Neutral | 5.0 | 5.0 | 4.9 |  |
| Important | 92.5 | 90.0 | 95.1 |  |
| **Labelling** |  |  |  |  |
| Unimportant | 9.9 | 5.0 | 14.6 | 0.298 |
| Neutral | 6.2 | 5.0 | 7.3 |  |
| Important | 83.9 | 90.0 | 70.1 |  |
| **Price** |  |  |  |  |
| Unimportant | 4.9 | 2.5 | 7.3 | 0.606 |
| Neutral | 7.4 | 7.5 | 7.3 |  |
| Important | 87.7 | 90.0 | 85.4 |  |
| **Child preference** |  |  |  |  |
| Unimportant | 8.6 | 17.5 |  | 0.006 |
| Neutral | 8.6 | 12.5 | 4.9 |  |
| Important | 82.8 | 70.0 | 95.1 |  |

*Supplementary Figure 1: Caregiver Perceptions about CPCFs Reasons why caregivers purchase CPCFs (reasons they strongly agree with)*

*
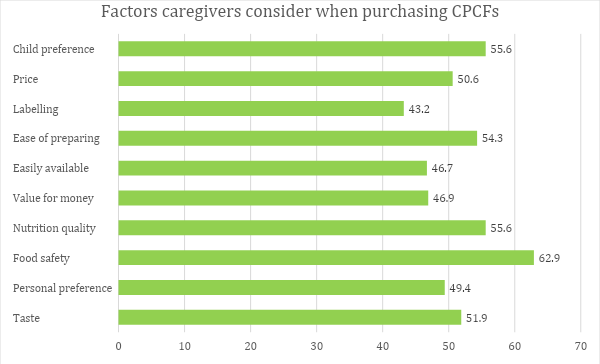
Supplementary Figure 2: Factors caregivers considered very important when purchasing CPCFs*


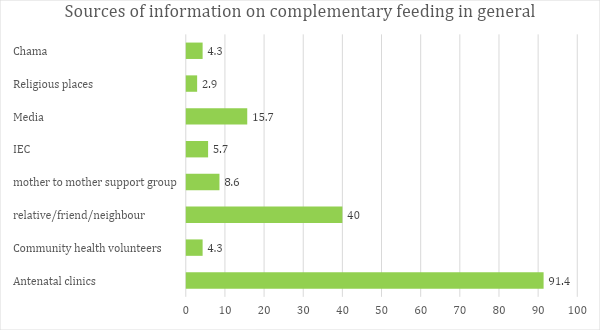


*Supplementary Figure 3: Sources of information on complementary feeding in general (n=70)*
